# CRISPR/Cas Tools for the Detection of *Borrelia* sensu lato in Human Samples

**DOI:** 10.3390/genes16101233

**Published:** 2025-10-18

**Authors:** Ermanno Nardon, Eros Azzalini, Dino Paladin, Diego Boscarino, Serena Bonin

**Affiliations:** 1 Department of Medical Sciences (DSM), University of Trieste, 34149 Trieste, Italy; enardon@units.it (E.N.); eazzalini@units.it (E.A.); 2 AB Analitica S.r.l., 35127 Padova, Italy; paladin@abanalitica.it (D.P.); boscarino@abanalitica.it (D.B.)

**Keywords:** *Borrelia*, Lyme disease, Cas12, PCR, detection

## Abstract

Background/Objectives: Lyme disease diagnosis remains challenging due to the limitations of current methods. While PCR-based assays are widely used, their sensitivity can be affected by sample type and the inhibition of host DNA. This study aimed to evaluate the feasibility and sensitivity of a CRISPR/Cas12-based detection system for *Borrelia burgdorferi* sensu lato, comparing its performance with real-time PCR. Methods: DNA from three *Borrelia* genospecies (*B. burgdorferi*, *B. garinii*, and *B. afzelii*) was amplified targeting the *OspA* gene. Detection was performed using a Cas12/crRNA system with a fluorescent ssDNA reporter. Sensitivity assays were conducted on serial dilutions of *Borrelia* DNA, with and without human genomic DNA, and results were compared with qPCR. Results: Direct detection of *Borrelia* DNA without amplification was not feasible. However, when combined with PCR, the Cas12/crRNA system reliably detected as few as 5 genome copies per reaction. End-point PCR extended to 60 cycles improved detection robustness for *B. garinii* and *B. afzelii*, although sensitivity decreased in the presence of human genomic DNA. Conclusions: The Cas12/crRNA-based system offers a sensitive and accessible alternative to qPCR, especially in settings lacking real-time PCR instrumentation. Future developments may include integration with isothermal amplification and microfluidic platforms to enhance direct detection capabilities.

## 1. Introduction

CRISPR gene systems (clustered regularly interspaced short palindromic repeats) have evolved in bacteria as adaptive immune mechanisms capable of identifying and degrading exogenous nucleic acid molecules through a set of CRISPR-associated (Cas) enzymes [1]. Target recognition relies on sequence complementarity between a crRNA (CRISPR RNA, or “guide RNA”) [2] and a target sequence flanked by a Protospacer Adjacent Motif (PAM), which is essential for the interaction between the target DNA and the Cas nuclease [1]. The crRNA can be synthetically designed to recognize sequences of interest for gene editing, therapeutic, or diagnostic applications [3].

The versatility of the CRISPR/Cas system is further enhanced by the availability of diverse Cas effector proteins, classified according to their evolutionary relationships, structural features, and mechanisms of action [4]. Notably, type V (Cas12) and type VI (Cas13) proteins are particularly promising for diagnostic use due to their ability to perform non-specific collateral cleavage (trans-cleavage) on ssDNA and ssRNA, respectively, upon target recognition by the guide RNA. This collateral nuclease activity is exploited to release a fluorescent signal by cleaving a synthetic ssDNA or ssRNA reporter molecule linked to a quencher [5,6].

Several methods have been developed for detecting RNA and DNA pathogens, typically coupled with nucleic acid amplification (isothermal or PCR), achieving attomolar sensitivity (~10^−18^ M, approximately one analyte molecule per µL) [4].

In this study, we evaluated the feasibility and sensitivity of a CRISPR/Cas-based approach for detecting the genome of *B. burgdorferi* sensu lato, the etiological agent of Lyme disease. Detection was performed on DNA extracted from three *Borrelia* strains, pre-amplified by real-time PCR, using a modified protocol previously applied for SNP detection [7]. We then compared the sensitivity of the Cas12/crRNA exonuclease system with real-time qPCR and conventional end-point PCR.

## 2. Materials and Methods

### 2.1. Borrelia Genospecies and Controls

DNA from three *Borrelia* genospecies, namely *B. burgdorferi* sensu stricto (B6), *Borrelia garinii*, and *Borrelia afzelii*, was obtained from the former Spirochete Laboratory of the University of Trieste and stored at −20 °C in aliquots at a concentration of 50,000 genomes/µL up to use. The control DNA consisted of a pool of genomic DNA extracted from peripheral blood of healthy donors, previously tested negative for *Borrelia* by qPCR, and stored at 4 °C at a concentration of 250 ng/µL up to use. The theoretical number of genome copies was based on the known genome size (in base pairs) of each *Borrelia* genospecies [8] and the average weight of a DNA base pair, which is approximately 1.096 × 10^−21^ g. To estimate the weight of a single genome in nanograms, the total number of base pairs was multiplied by the weight of one base pair and then converted to nanograms using the factor 10^9^:

Borrelia genome weight (ng) = Genome size (bp) × 1.096 × 10^−12^

The number of genome copies in a given DNA sample was then calculated by dividing the total DNA mass by the weight of the single *Borrelia* genome. Based on this estimation, the concentration of each serial dilution was inferred.

### 2.2. Direct Borrelia Detection by Cas12/crRNA/ssDNA Reporter 

The feasibility of detecting the *Borrelia* genome without prior PCR amplification was evaluated by directly adding *Borrelia* DNA to a reaction mixture containing Cas12, guide RNA, and a fluorophore. Serial dilutions of DNA from each *Borrelia* genospecies—*B. afzelii*, *B. garinii* and *B. burgdorferi* sensu stricto—ranging from 50,000 to 5 genome copies were prepared in 2 μL volumes and added to 18 μL of the previously described reaction mixture [7], outlined in Section 2.4.

### 2.3. qPCR and End-Point PCR

Both real-time and end-point PCR were carried out amplifying a 102 bp fragment of the *Borrelia* plasmidic *OspA* gene, using consensus primers as previously described [9]. For real-time PCR, the reaction was carried out in a final volume of 50 µL containing 1X JumpStart™ Taq Ready Mix (Merck-Sigma-Aldrich, St. Louis, MO, USA), 0.3 µM forward and reverse primers, and a TaqMan probe labeled with 6-FAM at the 5′ end and dual quenchers (ZEN™ and IABkFQ—Iowa Black™ Fluorescent Quencher) (Integrated DNA Technologies, Inc., Coralville, IA, USA) at a concentration of 0.2 µM. The final Mg^2+^ concentration was 5 mM. Amplification was performed on a BioRad CFX Opus 96 (Biorad, Hercules, CA, USA) instrument with the following conditions: 95 °C for 3 min, followed by 50 cycles of 95 °C for 20 s and 58 °C for 60 s.

End-point PCR was carried out in a final volume of 50 µL containing 1X PCR Buffer II (Thermo Fisher Scientific, Waltham, MA, USA), 5 mM Mg^2+^, 1.5 U Taq Gold Polymerase (Thermo Fisher Scientific), and 0.3 µM forward and reverse primers. Thermal cycling conditions were: 95 °C for 7 min and 30 s, followed by 60 cycles of 95 °C for 30 s, 58 °C for 60 s, and 72 °C for 30 s.

To determine the sensitivity of the amplification system, an aliquot of *Borrelia* DNA was thawed and serially diluted to obtain solutions containing between 5000 and 0.05 genomes/µL. One microliter of each dilution was used in the assay, either in the presence or absence of 4 µL of a pool of human DNA obtained from healthy donors (1000 ng DNA per assay). Each assay was performed in duplicate.

### 2.4. Detection of OspA Amplicon by Cas12/crRNA/ssDNA Reporter

The crRNA targeting a specific fragment within the *OspA* amplicon was designed using the online tool CRISPRdirect [10]. The software returned the same 20-nt long sequence for *B. burgdorferi* and *B. garinii*, and for *B. afzelii*, a sequence of the same length differing by only one nucleotide. The complete crRNA, which also included a 20-nt constant region specific to the Cas12 nuclease (loop domain), was synthesized by Integrated DNA Technologies. The complete crRNA sequence was 5′-UAAUUUCUACUAAGUGUAGAUCUUACAUGCUAUUAAGGCUA-3′ for *B. burdoferi* and *B. garinii*, and 5′-UAAUUUCUACUAAGUGUAGAUCUUGCAUGCUAUUAAGGCUA-3′ for *B. afzelii* (bases complementary to the target *OspA* sequence are underlined).

The detection mixture included 1X NEB 3 buffer (New England Biolabs, Ipswich, MA, USA), 500 nM of the newly synthesized crRNA, 250 nM recombinant Cas12a from *Lachnospiraceae bacterium* (Integrated DNA Technologies), 500 nM HEX-N12-BHQ1 reporter (Eurofins Genomics, Ebersberg, Germany), 10 U of DNase Inhibitor (Applied Biosystems), and 2 µL of amplification product in a final volume of 20 µL [7]. After adding the sample to the mixture, fluorescence was monitored for 30 min (at 30-s intervals) at 37 °C using the HEX channel (556 nm) on a BioRad CFX Opus 96 real-time thermocycler.

## 3. Results

### 3.1. Borrelia Detection by Cas12/crRNA/ssDNA Reporter Without Amplification

*Borrelia* DNA could not be detected without PCR amplification using the CRISPR-Cas12-based fluorescence assay. Although testing a wide range of DNA concentrations (from 50,000 to 5 genome copies), no fluorescence signal was observed above the negative control for any *Borrelia* genospecies, supporting that direct detection of *Borrelia* DNA using Cas12 without prior amplification is not feasible under the tested conditions.

### 3.2. Comparison Between Real-Time qPCR and Cas12/crRNA/ssDNA Detection System

**Figure 1:**
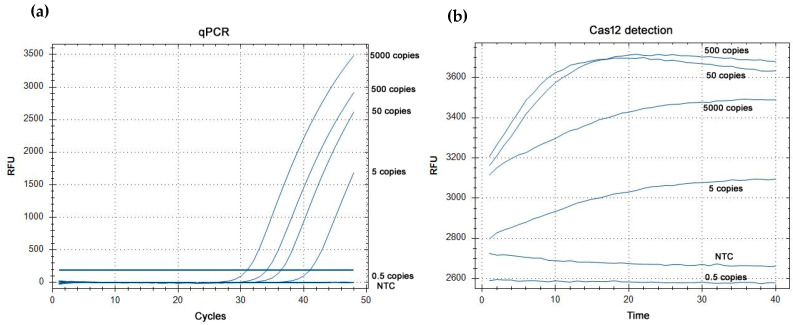
(**a**) Amplification curves by qPCR; (**b**) Fluorescence development kinetics of the Cas12/crRNA system.

The outcome of the real-time PCR expressed as positive/negative results and threshold cycle (Ct) was compared with the fluorescence development kinetics of the Cas12/crRNA/ssDNA system in a serial dilution of *Borrelia* DNA. For both systems the amplifications were carried out in a real-time PCR apparatus. Previously, it was confirmed that adding an amplification product containing a 6-FAM-labeled TaqMan probe did not induce any fluorescence overlap or bleed-through with the HEX reporter, when only the HEX channel was read. As a result, any observed increase in fluorescence during the Cas12 assays could be confidently attributed solely to the nuclease activity acting on the HEX-BHQ1 reporter. Assays without *Borrelia* DNA or human genomic DNA (i.e., No Template Control) consistently showed a stable fluorescence level over time. In contrast, assays that tested positive in real-time PCR exhibited a clear increase in fluorescence over time. Figure 1a clearly shows that all real-time assays showing exponential kinetics (even if delayed, i.e., with high Ct value) produced an unequivocal increase in fluorescence in the Cas12 assay (Figure 1b), resulting in saturation-type fluorescence kinetics. Generally, there was no correlation between plateau fluorescence levels and the initial quantity of the analyte under examination.

There was complete agreement (100%) between the outcome (positive/negative) of the real-time assay and the detection result obtained using the Cas12 system. The lower limit of detection (LOD) for both approaches was 5 genomes per assay for all three *Borrelia* genospecies. However, for *B. garinii*, this limit had poor reproducibility, as only one of the replicates tested positive (see Table 1). A complete agreement between the detection methods resulted even after the addition of 1000 ng of human genomic DNA. Regarding the detection sensitivity, it remained unchanged for *B. burgdorferi*, became poorly reproducible for *B. garinii*, and decreased to 50 genomes per assay for *B. afzelii* as reported in Table 1.

### 3.3. Comparison Between End-Point Amplification and Cas12/crRNA/ssDNA Detection System

To further enhance the detection sensitivity, traditional end-point amplification was employed, increasing the amplification cycles to 60 and combining the Cas12/crRNA-based detection. Sensitivity assays performed on serial dilutions of *Borrelia* DNA were compared with real-time amplification assessed in parallel on the same dilutions (see Table 2). End-point amplification improved the detection robustness of 5 genome copies per assay for *B. garinii* and *B. afzelii*, with both replicates testing positive. However, this advantage was lost in presence of human DNA, reducing the sensitivity for both genospecies to 50 copies/µL. In contrast, the limit of detection (LOD) of 5 copies per assay remained unchanged for *B. burgdorferi*, both in the absence and presence of human genomic DNA—matching the results obtained with qPCR.

## 4. Discussion

Direct diagnosis of Lyme disease remains challenging due to the complex clinical presentation and the limitations of current diagnostic tools. While culture-based identification is considered the gold standard for many extracellular bacteria, it is not practical for *Borrelia* spp. due to growth requirements. Culturing *Borrelia* necessitates highly specialized media and extended incubation periods, often several weeks, resulting in low sensitivity and limited clinical utility [11]. Molecular methods, particularly PCR-based assays are currently the most widely used tools for confirming *Borrelia* infection with direct methods [11,12]. Nonetheless, accurate diagnosis of Lyme disease remains challenging, even when using PCR, especially in blood samples due to the tropism of Lyme group *Borreliae* for tissues [13] and the transient spirochetemia [14]. Reported sensitivity of qPCR assays for Lyme group *Borrelia* detection varies between 10 and 40 copies per reaction depending on the sample type [15], the *B. burgdorferi* genospecies and the targeted marker [12]. In this study we evaluated whether a CRISPR/Cas12-based detection system could be used for Lyme group *Borrelia* and if this could enhance the sensitivity of the detection. Our findings indicate that direct detection of *Borrelia* using the Cas12/crRNA-based method was not feasible without a prior amplification step. This suggests that more sophisticated strategies, such as immobilization on chemically modified surfaces or integration into microfluidic platforms, may be required for effective direct detection [16]. Nevertheless, when combined with PCR amplification, the Cas12/crRNA-based system achieved a sensitivity comparable to our PCR based method, reliably detecting as few as 5 genome copies per reaction. Notably, extending end-point PCR to 60 cycles improved the robustness of detection for *B. garinii* and *B. afzelii* at low genome copy numbers. However, the presence of human genomic DNA appeared to reduce sensitivity, likely due to inhibitory effects as previously reported [17]. Nevertheless, in a subset of clinical cases, the Cas12/crRNA-based system confirmed the real-time PCR results.

It is important to note that genome copy numbers in our serial dilutions were theoretically estimated, which may have contributed to variability in detection at high dilutions, particularly for *B. afzelii* and *B. garinii*. The genome size of *Borrelia* is highly variable because of the variation in plasmid content across genospecies [18] and in different isolates [19] with possible over- or under-estimation of *Borrelia* concentrations in our serial dilutions. Additionally, minor differences in PCR efficiency among the three *Borrelia* genospecies cannot be excluded.

Our results demonstrate that the Cas12/crRNA-based detection system offers a viable alternative to qPCR, particularly in settings where real-time PCR instrumentation is unavailable. Detection can be performed using standard fluorescence microplate reader or via colorimetric readouts [7], following PCR amplification in an end-point thermocycler. Colorimetric CRISPR assays are especially promising for point-of-care applications, as they eliminate the need of specialized and expensive equipment. Future optimization could involve coupling loop-mediated isothermal amplification (LAMP) with Cas12/crRNA-based detection in the same tube to further simplify the workflow.

Overall, the analytical sensitivity achieved in this study using both qPCR and end-point PCR combined with Cas12/crRNA detection aligns with previously published data [4,7,20]. While CRISPR/Cas systems have been explored for gene silencing in *Borrelia* [21,22], to our knowledge, this is the first study to apply a Cas12/crRNA-based method for the detection of *Borrelia* DNA.

## Figures and Tables

**Table 1:** *Borrelia* DNA detection by *OspA* Cas12/crRNA vs. *OspA* qPCR real-time.

	*B. burgdorferi*				*B. garinii*				*B. afzelii*			
	w/o gDNA		+ ^a^ gDNA		w/o gDNA		+gDNA		w/o gDNA		+gDNA	
^b^ N° Genomes	qPCR (Ct)	Cas12 (RFU)	qPCR (Ct)	Cas12 (RFU)	qPCR (Ct)	Cas12 (RFU)	qPCR (Ct)	Cas12 (RFU)	qPCR (Ct)	Cas12 (RFU)	qPCR (Ct)	Cas12 (RFU)
**5000**	31.00	**3489**	30.24	**3713**	29.23	**3550**	29.77	**3464**	30.83	**3452**	30.22	**3375**
**5000**	30.83	**3711**	30.79	**3753**	29.31	**3516**	29.71	**3392**	30.98	**3593**	30.19	**3637**
**500**	34.12	**3680**	33.84	**3519**	33.07	**3508**	33.78	**3394**	35.08	**3354**	34.16	**3354**
**500**	34.09	**3443**	33.97	**3692**	32.82	**3435**	33.7	**3563**	34.23	**3596**	34.95	**3548**
**50**	38.71	**3634**	37.45	**3261**	36.82	**3365**	37.09	**2946**	37.33	**3842**	38.41	**3595**
**50**	38.67	**3096**	37.74	**3748**	37.01	**3293**	36.28	**3274**	38.54	**3601**	38.33	**3520**
**5**	N/A ^c^	2639	N/A	2663	39.63	**3322**	42.85	**3367**	39.29	**3670**	N/A	2672
**5**	41.09	**3111**	38.64	**3788**	38.68	**3118**	N/A	2690	N/A	2763	N/A	2687
**0.5**	N/A	2580	N/A	2637	N/A	2655	N/A	2648	N/A	2623	N/A	2680
**0.5**	N/A	2666	N/A	2647	N/A	2652	N/A	2657	N/A	2662	N/A	2702
**0**	N/A	2661	N/A	2562	N/A	2698	N/A	2682	N/A	2698	N/A	2583

^a^ Human genomic DNA; ^b^ number of *Borrelia* genomes per assay; ^c^ N/A non-available. Bold characters indicate the RFUs of positive samples.

**Table 2:** *OspA Borrelia* detection by Cas12/crRNA/end-point PCR vs. qPCR real-time.

*B. burgdorferi*				*B. garinii*			*B. afzelii*		
N° Genomes	qPCR (Ct)	End-Point (RFU)	End-Point +DNA (RFU)	qPCR (Ct)	End-Point (RFU)	End-Point +DNA (RFU)	qPCR (Ct)	End-Point (RFU)	End-Point +DNA (RFU)
**500**	30.57	**7017**	**7779**	33.16	**8459**	**8380**	33.25	**8080**	**8565**
**500**	30.41	**7690**	**6163**	33.16	**7777**	**8053**	33.05	**8541**	**7582**
**50**	34.28	**7124**	**7015**	36.05	**8872**	**8606**	37.00	**7529**	**8813**
**50**	34.54	**7190**	**6317**	37.11	**8491**	**7902**	36.61	**8682**	**8182**
**5**	38.02	**6496**	**6777**	39.9	**8190**	**8156**	38.35	**8622**	**8650**
**5**	38.27	**6732**	**6150**	39.76	**8052**	**7654**	39.37	**8124**	**8248**
**0.5**	40.11	**7757**	**7171**	49.84	**8577**	3478	40.51	**7798**	3326
**0.5**	N/A	3302	3150	N/A	**7846**	3331	N/A	**8494**	3550
**0.05**	N/A	3091	3093	N/A	3374	3459	N/A	3250	3412
**0.05**	N/A	3153	3042	N/A	3423	3522	N/A	3438	3471
**0**	N/A	2950	3150	N/A	3370	2933	N/A	3279	3495

Bold characters indicate the RFUs of positive samples, N/A non-available.

## Data Availability

Data supporting the study are included in the article.

## Abbreviations

The following abbreviations are used in this manuscript:

CRISPR	Clustered regularly interspaced short palindromic repeats
Cas	CRISPR-associated
PAM	Protospacer Adjacent Motif
crRNA	CRISPR RNA or guide RNA
SNP	Single Nucleotide Polymorphism
qPCR	Quantitative Polymerase Chain Reaction
